# Age-Adjusted Mortality Trends in Acute Tubulointerstitial Nephritis by Gender, Race, and Census Region in the United States: A CDC-WONDER Study, 1999–2020

**DOI:** 10.3390/jcm15041501

**Published:** 2026-02-14

**Authors:** Abdallah Ibrahim Abujlambo, Muhammad Ali Khan, Hiba Hamdar, Bassam G. Abu Jawdeh

**Affiliations:** 1 Department of ICU, Al-Shifa Hospital, Gaza Strip, Palestine; abdallah.abojlambo@gmail.com; 2 Division of Nephrology and Hypertension, Mayo Clinic Arizona, 577 E Mayo Blvd., Phoenix, AZ 85054, USA; 3 Plovdiv Medical University, 4002 Plovdiv, Bulgaria; hamdarhiba95@gmail.com

**Keywords:** acute tubulointerstitial nephritis, mortality trends, age-adjusted mortality rates, racial disparities, geographic variation, CDC WONDER, United States

## Abstract

**Background:** Acute tubulointerstitial nephritis (ATIN) is a significant yet under-monitored cause of U.S. mortality, particularly among the elderly. This study anrackalyzed national trends and demographic disparities in age-adjusted mortality rates (AAMRs) from 1999 to 2020 to identify high-risk populations and inform public health policy. **Methods:** Using the CDC WONDER database, we conducted a retrospective analysis of 6872 ATIN-related deaths. AAMRs (per 100,000) were stratified by sex, race, and census region. Temporal shifts were quantified using Joinpoint regression to determine annual percentage changes (APC) and 95% confidence intervals (CIs). **Results:** The analysis revealed a distinct “V-shaped” mortality trend across the 22-year period. Following an initial decline from 1999 to 2013, AAMRs rose sharply through 2020. Males experienced a slightly steeper recent increase (9.90%) compared to females (9.50%). While Black/African American individuals initially had higher mortality rates, a significant surge in deaths among Non-Hispanic White individuals after 2013 (APC 10.42%) led to a convergence of mortality rates between the two groups by 2020. Geographically, the Midwest (APC 12.08%) and the South saw the most pronounced recent increases, whereas the West showed a sustained upward trend beginning as early as 2008. **Conclusions:** There has been a concerning reversal in ATIN-related mortality trends in the United States over the last decade. The convergence of racial mortality rates and significant regional variations suggest that shifting healthcare access, environmental factors, or medication exposure patterns (such as polypharmacy) warrant urgent investigation to mitigate this rising public health burden.

## 1. Introduction

Acute tubulointerstitial nephritis (ATIN) is an important cause of kidney injury and is characterized by inflammatory involvement of the renal interstitium with associated impairment of renal function [1]. ATIN most commonly results from drug-induced hypersensitivity reactions, particularly related to antibiotics, nonsteroidal anti-inflammatory drugs (NSAIDs), and proton pump inhibitors (PPIs) [2,3]. Less frequently, ATIN may arise secondary to infections, autoimmune diseases, or idiopathic etiologies [1,2]. Although ATIN accounts for approximately 15–27% of acute kidney injury (AKI) cases requiring kidney biopsy, its true incidence and prevalence are likely underestimated due to heterogeneous clinical presentations and reliance on invasive diagnostic methods [1]. Recent evidence suggests that the prevalence of ATIN has increased over the past 16 years, particularly among older adults (>65 years) [4], with the rising burden over the last decade largely attributed to widespread and prolonged PPI use [5]. These trends underscore the growing clinical significance of ATIN.

While ATIN is often considered a reversible condition when diagnosed early and managed by discontinuation of the offending agent, delayed detection may result in irreversible kidney injury with progression to chronic kidney damage and, in severe cases, End-Stage Renal Disease (ESRD) requiring hemodialysis [6]. In addition, ATIN is associated with increased morbidity and mortality among older adults [4]. Progressive decline in glomerular filtration rate (GFR) occurs in up to 40% of cases of drug-induced ATIN, leading to the development of chronic kidney disease (CKD) [2,3]. Several studies have highlighted the impact of sociodemographic, environmental, and demographic factors on ATIN susceptibility and outcomes; however, these associations have not been adequately examined at the national level [7,8]. Racial differences are frequently observed in the clinical outcomes of multiple medical conditions in the United States [7]. Race represents a key demographic factor contributing to disparities in AKI, with Black individuals experiencing a higher risk of AKI and a greater likelihood of progression to ESRD compared with their White counterparts, often attributed to differences in healthcare access, genetic factors, and comorbidity burden [9]. Moreover, regional disparities in healthcare delivery, physician availability, environmental exposures, and socioeconomic conditions further complicate kidney disease outcomes across the United States [8,10]. Despite this, substantial gaps remain in the epidemiologic understanding of long-term mortality patterns associated with ATIN, particularly regarding variations by sex, race, and census region. Comprehensive epidemiological surveillance is therefore essential to identify temporal patterns and inform targeted prevention and management strategies.

This study aimed to characterize national mortality trends related to acute tubulointerstitial nephritis (ATIN) from 1999 to 2020 using data from the CDC WONDER database. Specifically, we sought to analyze age-adjusted mortality rates stratified by sex, race, and census region to identify temporal patterns and demographic disparities in ATIN-related mortality.

## 2. Methodology

### 2.1. Data Sources

Mortality data related to ATIN were obtained from the CDC WONDER database using Multiple Cause of Death (MCOD) records, which provide publicly accessible, deidentified U.S. death certificate data [11,12]. ATIN was identified using the International Classification of Diseases, 10th Revision Clinical Modification (ICD-10-CM) code N10, listed as either the primary or a contributing cause of death [13]. This analysis was conducted in accordance with the STROBE (Strengthening the Reporting of Observational Studies in Epidemiology) guidelines [14]. Institutional Review Board approval was not required because the study used publicly available, deidentified data.

### 2.2. Study Variables Extraction

Age-adjusted mortality rate (AAMRs) data for ATIN from 1999 to 2020 were extracted for analysis. Stratified analyses were performed across key demographic and geographic variables to evaluate disparities in mortality patterns. Demographic variables included gender and race/ethnicity, while geographic variables included U.S. census regions. Race/ethnicity was classified into non-Hispanic (NH) White, NH Black, NH Asian or Pacific Islander, and Hispanic categories according to the U.S. Office of Management and Budget guidelines and CDC WONDER classifications [15]. Geographic regions were defined using U.S. Census Bureau classifications and stratified into Northeast, Midwest, South, and West [16]. Geographic visualization was performed at the level of U.S. Census divisions to provide greater spatial granularity. Census divisions are standardized subregional groupings nested within the four Census regions and allow for more detailed visualization of regional heterogeneity. Census divisions were used solely for mapping purposes and did not alter the regional categorization applied in statistical analyses [16].

### 2.3. Statistical Analysis

**Figure 1:**
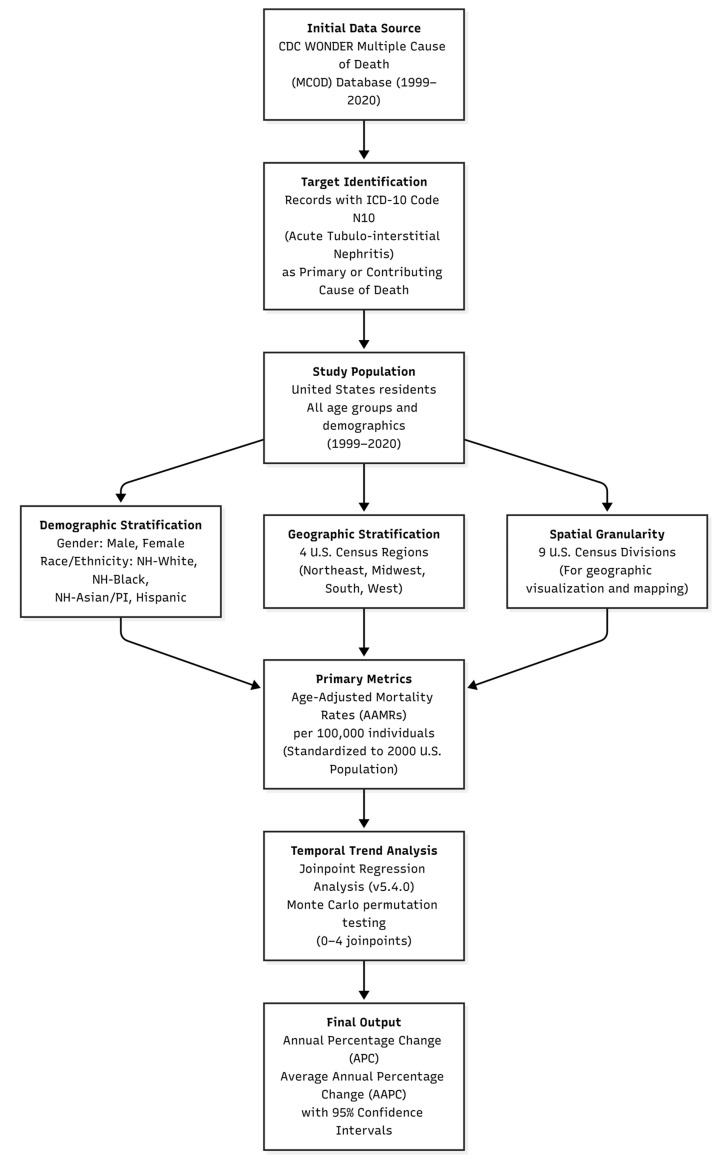
Flowchart of the study methodology outlining the data extraction process from the CDC WONDER database, demographic and geographic stratification, and the statistical framework used for Joinpoint regression analysis of ATIN-related mortality (1999–2020).

Age-adjusted mortality rates (AAMRs) per 100,000 individuals were calculated using the year 2000 U.S. standard population as the reference [17]. Statistical significance was defined as *p* < 0.05, with corresponding 95% confidence intervals (CIs). Joinpoint regression analysis was performed using the Joinpoint Regression Program (version 5.4.0, National Cancer Institute, Rockville, MD, USA) to identify significant changes in mortality trends over time by fitting a series of joined straight lines to log-transformed AAMRs and estimating annual percentage changes (APCs) [18]. Changes in slope were interpreted as shifts in mortality trends over time. Permutation testing with Monte Carlo resampling (4499 permutations), allowing 0 to 4 joinpoints, was used to estimate APCs and their 95% CIs at each joinpoint. Overall temporal trends were summarized using average annual percent changes (AAPCs), calculated as weighted averages of APCs across the study period, with statistical significance defined as *p* < 0.05. (See Figure 1).

## 3. Results

### 3.1. Overall Data

From 1999 to 2020, a total of 6872 deaths due to ATIN were recorded among adults aged ≥25 years. Over the two-decade period, overall AAMRs demonstrated a significant long-term decline, with an AAPC of −1.62 (95% CI: 0.33–2.93; *p* = 0.013). Joinpoint analysis identified two inflection points. AAMRs declined significantly from 1999 to 2013 (APC −1.88; 95% CI: −3.21 to −0.53), followed by a marked increase from 2013 to 2020 (APC 9.00; 95% CI: 5.69–12.41).

AAMRs varied over time across sex, race, and geographic region. Overall AAMRs were 0.15 per 100,000 in 1999 and 0.21 per 100,000 in 2020. Among females, AAMRs remained relatively stable from 1999 to 2013 (approximately 0.14) before increasing sharply to 0.26 in 2020. A similar pattern was observed in males, with AAMRs increasing from 0.13 in 1999 to 0.18 in 2020.

**Figure 2:**
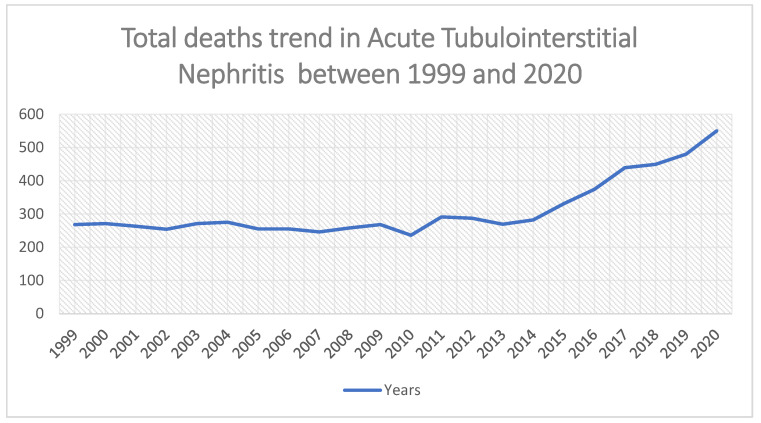
Total number of deaths due to ATIN from 1999 to 2020 across the U.S.

By race, AAMRs were higher among Black individuals (0.28 in 1999) compared with White individuals (0.13 in 1999). This disparity narrowed over time, with both groups exhibiting comparable AAMRs of 0.23 in 2020. Regionally, AAMRs remained consistently highest in the South, increasing from 0.22 in 1999 to 0.26 in 2020. The Northeast had the lowest AAMR in 2020 (0.11), while the Midwest and West demonstrated comparable rising trends over the study period (Figure 2, Supplementary Tables S1 and S2).

### 3.2. Gender Disparities

**Figure 3:**
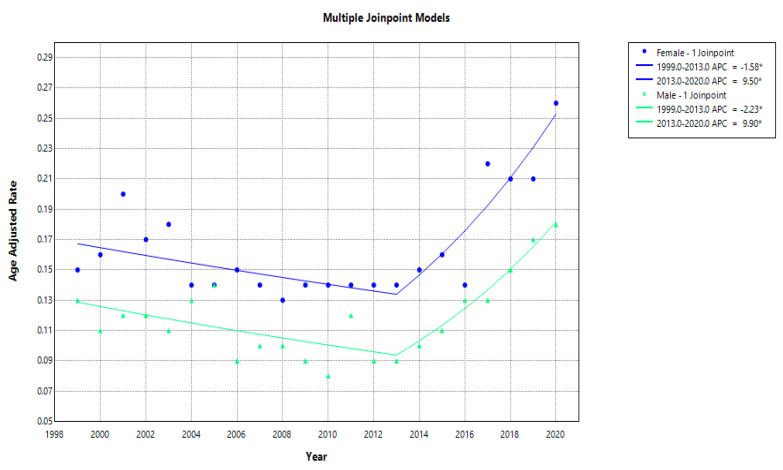
Gender trends and disparities in AAMRs per 100,000 for ATIN from 1999 to 2020. * Indicates statistical significance (*p* < 0.05).

The overall AAMRs increased in both males and females from 1999 to 2020, with AAPCs of 1.99 (95% CI: 0.55–3.45, *p* = 0.006) and 1.66 (95% CI: 0.004–3.33, *p* = 0.049), respectively. In males, there is a single inflection point; the AAMRs trend was reversed with (APC −2.23*), suggesting a greater initial improvement compared to females. In comparison, there was a significant rise in mortality between 2013 and 2020 (APC 9.90*). The same pattern happened in females with a statistically significant decrease in AAMRs with APC (−1.58*); however, this trend reversed between 2013 and 2020, with a sharp increase (APC 9.50*) (Figure 3, Supplementary Table S3).

### 3.3. Racial Disparities

**Figure 4:**
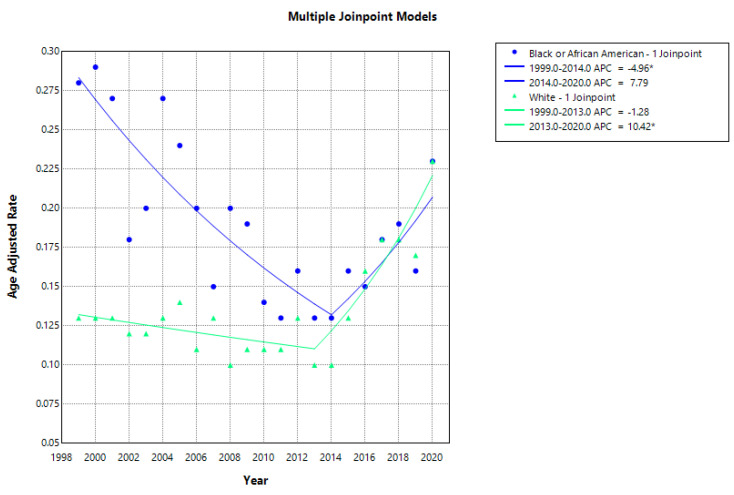
Racial trends and disparities in AAMR per 100,000 for ATIN from 1999 to 2020. * Indicates statistical significance (*p* < 0.05).

Among NH White individuals, overall AAMRs increased significantly over the study period, with an AAPC of 2.48 (95% CI: 1.03–3.94; *p* = 0.0007). In contrast, NH Black individuals experienced a non-significant overall decline in AAMRs (AAPC −1.48; 95% CI: −3.80 to 0.89; *p* = 0.219). Joinpoint analysis identified a single inflection point among NH Black individuals, with a significant decline in AAMRs from 1999 to 2013 (APC −4.96), followed by a non-significant increase from 2013 to 2020 (APC 7.79; *p* = 0.051). Among NH White individuals, AAMRs showed a non-significant decline between 1999 and 2013 (APC −1.28), followed by a pronounced increase between 2013 and 2020 (APC 10.42; *p* < 0.000001), exceeding the rate of increase observed among NH Black individuals. Due to insufficient reliable data, NH Asian and Hispanic populations were excluded from this analysis (Figure 4, Supplementary Table S4).

### 3.4. Census Region Disparities

Overall AAPCs indicated non-significant increases in AAMRs across all U.S. census regions. Nevertheless, distinct regional disparities in ATIN-related mortality were observed. In the Northeast, mortality demonstrated a biphasic pattern, with a non-significant decline from 1999 to 2013 (APC −2.07), followed by a significant increase from 2013 to 2020 (APC 9.68; *p* = 0.004). A similar but more pronounced pattern was observed in the Midwest, where AAMRs declined modestly and non-significantly between 1999 and 2014 (APC −1.23) before rising sharply thereafter, representing the steepest increase among all regions (APC 12.08; *p* < 0.01).

**Figure 5:**
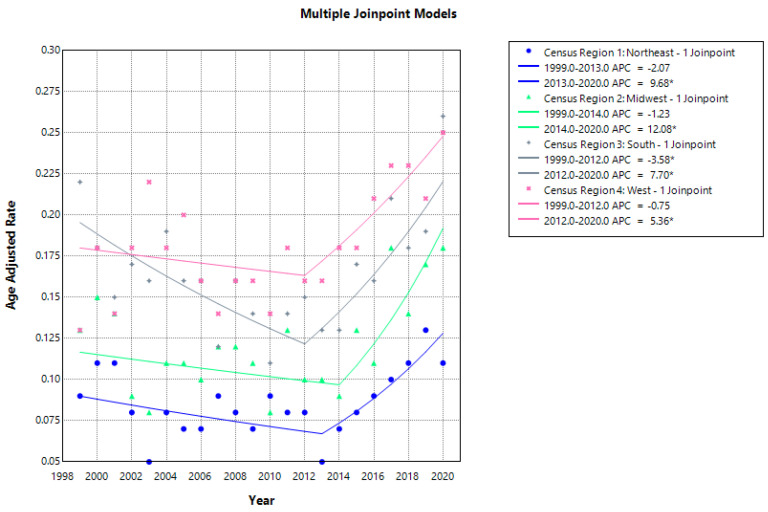
Regional trends and disparities in AAMR per 100,000 for ATIN across census regions from 1999 to 2020. * Indicates statistical significance (*p* < 0.05).

**Figure 6:**
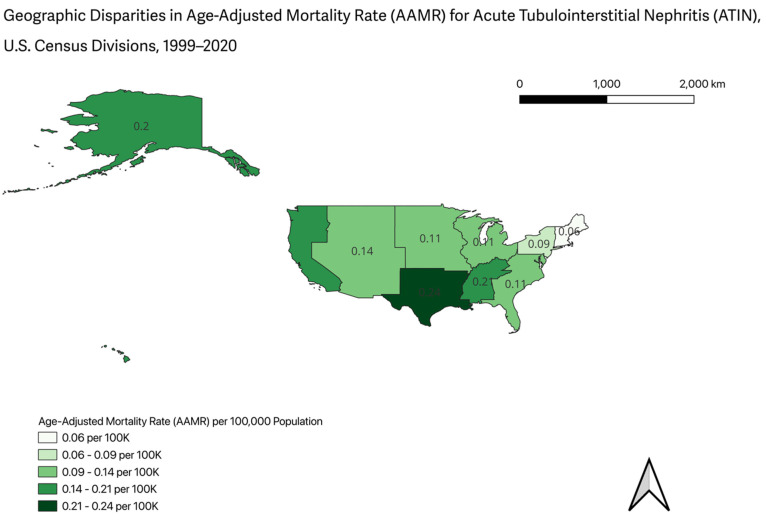
Geographic Disparities in Age-Adjusted Mortality Rate (AAMR) for Acute Tubulointerstitial nephritis (ATIN), U.S. Census Divisions, 1999–2020.

The South followed a comparable trajectory, with a significant decline in mortality from 1999 to 2012 (APC −3.58), followed by a marked increase from 2013 to 2020 (APC 7.70; *p* = 0.0001). In contrast, the West exhibited a distinct temporal pattern, with a non-significant increase in AAMRs from 1999 to 2003 (APC 10.57), followed by a non-significant decline from 2003 to 2008 (APC −7.17), and a subsequent significant increase from 2008 to 2020 (APC 4.41; *p* < 0.00008) (Figure 5 and Figure 6, Supplementary Tables S5 and S6).

## 4. Discussion

This study examined 20-year trends (1999–2020) in ATIN-related mortality in the United States and identified persistent demographic and regional disparities. Although mortality rates initially declined, a concerning reversal occurred after 2013, affecting both sexes, NH White individuals, and multiple census regions. Post-2013, the South consistently exhibited the highest mortality burden, while the Midwest experienced the steepest increase in mortality (APC = 12.08%), followed by the Northeast and South.

The potential explanation for this finding is a change in the attitude of healthcare workers with early introduction of antibiotics [19] and, later increasing prescription of nephrotoxic agents particularly PPIs over the last decade [5]. Additional contributors include widespread use of NSAIDS and immune checkpoint inhibitors (ICI), especially among older adults [1,2,20,21]. Hossain et al. (2017) also identified the opioid crisis as a key population-level change in the United States in recent years [22]. Opioid misuse has emerged as a major public health burden in the United States, with more than 12.5 million individuals reporting non-medical opioid use [23]. Opioid-related toxicity, including heroin overdose, can precipitate AKI that may go unrecognized, leading to potentially preventable progression to advanced CKD [23]. However, it is important to emphasize that the present study is ecological and descriptive in nature and lacks individual-level data on medication exposure. Consequently, these associations should be interpreted as hypothesis-generating rather than causal. Although prior studies have documented increasing use of PPIs, NSAIDs, and ICIs during the study period, we were unable to directly correlate prescription trends with ATIN-related mortality or confirm temporal alignment with the observed inflection point after 2013. In addition, definitive diagnosis of ATIN typically requires renal biopsy, which is often not pursued in routine clinical practice. Coupled with frequently nonspecific clinical presentations, particularly among populations with limited access to healthcare, this diagnostic uncertainty may delay treatment and increase the risk of mortality [1,3].

The increase in ATIN-related mortality after 2013 among both males and females may suggest the influence of system-level factors, including polypharmacy, greater exposure to nephrotoxic medications, and expanding use of chemotherapeutic agents, rather than sex-specific effects alone [24]. Men have a higher lifetime risk of developing ESRD and tend to experience faster progression of CKD compared with women. This could be related to the protective effects of estrogen in women as well as higher-risk behaviors among men, such as delayed healthcare seeking, increased NSAID use, and greater exposure to nephrotoxic substances [25].

The declining mortality trends observed among both NH White and NH Black populations from 1999 to 2013 may reflect the implementation of targeted public health interventions, improved healthcare access, and increased awareness of chronic disease prevention, particularly among high-risk groups [9]. After 2013, the White–Black mortality gap narrowed as mortality rates rose more sharply among White individuals, potentially reflecting increased medication-associated ATIN [4,5]. These disparities may also arise from complex interactions involving social determinants of health, including systemic healthcare inequities, socioeconomic disadvantage, and delayed referral to nephrology care, which disproportionately increase the risk of AKI and CKD among Black individuals [8,9,26,27]. In addition, biological factors, including genetic susceptibility such as APOL1 risk alleles, further increase vulnerability to kidney injury in African American populations [8,9,28]. Consistent with prior reports, NH Black individuals have higher incidence rates of AKI [9,29], and AKI has been associated with a fourfold increase in mortality, underscoring the importance of early recognition and monitoring of ATIN in hospitalized patients [30].

These findings are consistent with national data demonstrating a higher prevalence of CKD in southern U.S. regions, where poverty rates are also elevated [31]. Older adults in these areas have a greater burden of CKD and, consequently, increased susceptibility to ATIN, which may translate into higher mortality in the setting of polypharmacy, drug-induced nephrotoxicity, and unrecognized AKI [31]. Rural populations in the Midwest and South face substantial barriers to specialty care, including limited access to nephrology services and delayed referrals, which adversely affect outcomes among patients with AKI and CKD [10]. In contrast, data from the Medical Expenditure Panel Survey indicate that individuals in the Northeast are more likely to have a usual source of care and broader healthcare access, facilitating earlier diagnosis of ATIN and timely specialist involvement [32]. Additional evidence supports persistent geographic disparities in nephrotoxic medication exposure; for example, rural populations in Virginia have been shown to have higher NSAIDs use compared with non-rural populations [33].

### Limitations

This study has several limitations inherent to the use of retrospective administrative mortality data. First, diagnostic misclassification and potential “diagnostic drift” must be considered. Because acute tubulointerstitial nephritis (ATIN) is ideally confirmed by renal biopsy, reliance on clinician-reported death certificate data without mandatory pathological verification may lead to underdiagnosis or misclassification, particularly in earlier years. Many cases may have been coded as nonspecific acute kidney injury, and the observed increase in mortality may partially reflect improved clinical recognition and coding practices over time rather than a true rise in incidence.

Second, there are constraints regarding population generalizability due to statistical reliability thresholds. Hispanic and Asian populations were excluded from the primary Joinpoint trend analysis. Per National Center for Health Statistics (NCHS) standards, data are considered suppressed or unreliable when annual death counts are fewer than 20 or the Relative Standard Error (RSE) exceeds 23%. While this prevents the modeling of stable temporal trends for these groups, the low frequency of ATIN-coded deaths in these large demographic blocks is a finding in itself, potentially suggesting lower incidence, different environmental exposures, or significant underdiagnosis in these communities. Additionally, the CDC WONDER database lacks individual-level clinical detail, precluding adjustment for specific medication exposures, comorbidities, and socioeconomic factors known to influence ATIN risk and outcomes. These limitations highlight the need for future prospective studies with greater clinical granularity.

Finally, mortality estimates for 2020 should be interpreted with caution, as COVID-19–related acute kidney injury, healthcare disruptions, and changes in cause-of-death reporting practices during the pandemic may have influenced ATIN attribution and mortality trends.

## 5. Conclusions

This study demonstrates an initial decline in ATIN-related mortality in the United States, followed by a concerning reversal in national mortality trends after 2013. These patterns likely reflect increasing exposure to nephrotoxic medications, the ongoing opioid crisis, and the growing burden of CKD. Public health efforts should prioritize early recognition of drug-induced AKI, safer prescribing practices, and equitable access to healthcare resources across racial groups and census regions to reduce ATIN-related mortality.

## Data Availability

This study utilized publicly available mortality data from the Centers for Disease Control and Prevention (CDC) Wide-ranging Online Data for Epidemiologic Research (WONDER) database (https://wonder.cdc.gov/, accessed on 4 December 2025). All analyses were conducted using these publicly accessible datasets, and no additional data were generated.

## Supplementary Materials

The following supporting information can be downloaded at: https://www.mdpi.com/article/10.3390/jcm15041501/s1, Supplementary Table S1. Age-Adjusted Mortality Rates (AAMRs) per 100,000 Population Overall and Stratified by Sex, Race (White, Black), and Census Region, 1999–2020. Supplementary Table S2. Annual Percent Change (APC) in Overall Age-Adjusted Mortality Rates Due to Acute Tubulointerstitial Nephritis (ATIN), 1999–2020. Supplementary Table S3. Annual Percent Change (APC) in Age-Adjusted Mortality Rates Due to Acute Tubulointerstitial Nephritis (ATIN), Stratified by Sex, 1999–2020. Table S4. Annual Percent Change (APC) in Age-Adjusted Mortality Rates Due to Acute Tubulointerstitial Nephritis (ATIN), Stratified by Race, 1999–2020. Supplementary Table S5. Average Annual Percent Change (AAPC) in Age-Adjusted Mortality Rates per 100,000 Population by Census Region, 1999–2020. Supplementary Table S6. Annual Percent Change (APC) in Age-Adjusted Mortality Rates per 100,000 Population by Census Region, 1999–2020.

## Abbreviations

The following abbreviations are used in this manuscript:

AAMR	Age-Adjusted Mortality Rate
AAPC	Average Annual Percent Change
AKI	Acute Kidney Injury
APC	Annual Percent Change
APOL1	Apolipoprotein L1
ATIN	Acute Tubulointerstitial Nephritis
CDC	Centers for Disease Control and Prevention
CI	Confidence Interval
CKD	Chronic Kidney Disease
ESRD	End-Stage Renal Disease
GFR	Glomerular Filtration Rate
ICD-10-CM	International Classification of Diseases, 10th Revision, Clinical Modification
ICI	Immune Checkpoint Inhibitors
MCOD	Multiple Cause of Death
NH	Non-Hispanic
NSAIDs	Nonsteroidal Anti-Inflammatory Drugs
PPIs	Proton Pump Inhibitors
STROBE	Strengthening the Reporting of Observational Studies in Epidemiology
U.S.	United States
WONDER	Wide-ranging Online Data for Epidemiologic Research
