# Supplementary material for: Age-Adjusted Mortality Trends in Acute Tubulointerstitial Nephritis by Gender, Race, and Census Region in the United States: A CDC-WONDER Study, 1999–2020
Source: J Clin Med. 2026 Feb 14;15(4):1501. doi: 10.3390/jcm15041501 (PMC12941581; doi:10.3390/jcm15041501)
Supplement: Supplementary file 1 [file jcm-15-01501-s001.zip › jcm-4125816-supplementary.pdf]

Supplementary Table S1: Age-Adjusted Mortality Rates (AAMRs) per 100,000 population overall, and stratified by sex, race (e.g., White, Black), and census region from 1999-2020.

Year	AAMRs (Male)	AAMRs (Female)	AAMRs (White)	AAMRs (Black)	AAMRs (Northeast)	AAMRs (Midwest)	AAMRs (South)	AAMRs (West)	AAMRs (Total)
**1999**	0.13 (0.10–0.16)	0.15 (0.13–0.18)	0.13 (0.11–0.15)	0.28 (0.20–0.38)	0.09 (0.06–0.14)	0.13 (0.09–0.16)	0.22 (0.18–0.26)	0.13 (0.09–0.17)	0.14 (0.12–0.16)
**2000**	0.11 (0.09–0.14)	0.16 (0.14–0.19)	0.13 (0.12–0.15)	0.29 (0.21–0.39)	0.11 (0.07–0.15)	0.15 (0.11–0.19)	0.18 (0.15–0.22)	0.18 (0.14–0.23)	0.14 (0.12–0.15)
**2001**	0.12 (0.09–0.15)	0.20 (0.17–0.23)	0.13 (0.11–0.14)	0.27 (0.19–0.36)	0.11 (0.07–0.15)	0.14 (0.11–0.18)	0.15 (0.12–0.18)	0.14 (0.11–0.19)	0.15 (0.13–0.17)
**2002**	0.12 (0.10–0.15)	0.17 (0.14–0.20)	0.12 (0.11–0.14)	0.18 (0.12–0.26)	0.08 (0.05–0.12)	0.09 (0.06–0.12)	0.17 (0.13–0.20)	0.18 (0.14–0.23)	0.12 (0.11–0.14)
**2003**	0.11 (0.09–0.14)	0.18 (0.15–0.21)	0.12 (0.10–0.14)	0.20 (0.14–0.28)	0.05 (0.03–0.08)	0.08 (0.05–0.11)	0.16 (0.13–0.19)	0.22 (0.17–0.27)	0.15 (0.13–0.17)
**2004**	0.13 (0.10–0.16)	0.14 (0.12–0.16)	0.13 (0.11–0.15)	0.27 (0.20–0.36)	0.08 (0.05–0.12)	0.11 (0.08–0.15)	0.19 (0.16–0.23)	0.18 (0.14–0.23)	0.13 (0.12–0.15)
**2005**	0.14 (0.11–0.17)	0.14 (0.12–0.17)	0.14 (0.12–0.16)	0.24 (0.17–0.32)	0.07 (0.04–0.10)	0.11 (0.08–0.14)	0.16 (0.13–0.19)	0.20 (0.15–0.24)	0.14 (0.12–0.16)
**2006**	0.09 (0.07–0.12)	0.15 (0.13–0.18)	0.11 (0.10–0.13)	0.20 (0.14–0.28)	0.07 (0.05–0.11)	0.10 (0.07–0.13)	0.16 (0.13–0.19)	0.16 (0.12–0.21)	0.14 (0.12–0.16)
**2007**	0.10 (0.08–0.12)	0.14 (0.11–0.16)	0.13 (0.11–0.14)	0.15 (0.10–0.22)	0.09 (0.06–0.13)	0.12 (0.09–0.16)	0.12 (0.10–0.15)	0.14 (0.11–0.18)	0.14 (0.12–0.15)

**2008**	0.10 (0.08–0.12)	0.13 (0.11–0.16)	0.10 (0.09–0.12)	0.20 (0.14–0.28)	0.08 (0.05–0.13)	0.12 (0.09–0.16)	0.16 (0.12–0.19)	0.16 (0.12–0.20)	0.11 (0.09–0.12)
**2009**	0.09 (0.08–0.12)	0.14 (0.12–0.17)	0.11 (0.09–0.12)	0.19 (0.13–0.26)	0.07 (0.05–0.10)	0.11 (0.08–0.15)	0.14 (0.11–0.17)	0.16 (0.12–0.20)	0.13 (0.12–0.15)
**2010**	0.08 (0.06–0.10)	0.14 (0.11–0.16)	0.11 (0.10–0.13)	0.14 (0.09–0.20)	0.09 (0.06–0.13)	0.08 (0.06–0.12)	0.11 (0.09–0.14)	0.14 (0.11–0.18)	0.10 (0.09–0.12)
**2011**	0.12 (0.10–0.15)	0.14 (0.12–0.17)	0.11 (0.10–0.13)	0.13 (0.09–0.18)	0.08 (0.05–0.12)	0.13 (0.10–0.16)	0.14 (0.11–0.17)	0.18 (0.15–0.23)	0.12 (0.10–0.13)
**2012**	0.09 (0.07–0.11)	0.14 (0.12–0.16)	0.13 (0.11–0.15)	0.16 (0.11–0.23)	0.08 (0.06–0.11)	0.10 (0.07–0.13)	0.15 (0.12–0.18)	0.16 (0.13–0.20)	0.11 (0.10–0.12)
**2013**	0.09 (0.07–0.11)	0.14 (0.12–0.16)	0.10 (0.09–0.12)	0.13 (0.08–0.19)	0.05 (0.03–0.07)	0.10 (0.07–0.14)	0.13 (0.10–0.16)	0.16 (0.12–0.20)	0.10 (0.09–0.11)
**2014**	0.10 (0.08–0.12)	0.15 (0.13–0.17)	0.10 (0.09–0.12)	0.13 (0.09–0.19)	0.07 (0.05–0.10)	0.09 (0.07–0.12)	0.13 (0.10–0.16)	0.18 (0.14–0.22)	0.13 (0.11–0.14)
**2015**	0.11 (0.09–0.13)	0.16 (0.14–0.19)	0.13 (0.11–0.14)	0.16 (0.11–0.22)	0.08 (0.05–0.11)	0.13 (0.10–0.16)	0.17 (0.14–0.20)	0.18 (0.14–0.22)	0.13 (0.11–0.14)
**2016**	0.13 (0.11–0.16)	0.14 (0.12–0.16)	0.16 (0.14–0.18)	0.15 (0.11–0.21)	0.09 (0.06–0.13)	0.11 (0.08–0.15)	0.16 (0.13–0.19)	0.21 (0.17–0.25)	0.14 (0.12–0.15)
**2017**	0.13 (0.10–0.15)	0.22 (0.19–0.25)	0.18 (0.16–0.20)	0.18 (0.13–0.24)	0.10 (0.07–0.14)	0.18 (0.14–0.22)	0.21 (0.18–0.25)	0.23 (0.18–0.27)	0.18 (0.16–0.20)

**2018**	0.15 (0.13–0.18)	0.21 (0.18–0.24)	0.18 (0.16–0.20)	0.19 (0.14–0.26)	0.11 (0.08–0.16)	0.14 (0.11–0.17)	0.18 (0.15–0.20)	0.23 (0.18–0.27)	0.18 (0.16–0.20)
**2019**	0.17 (0.15–0.20)	0.21 (0.18–0.24)	0.17 (0.15–0.19)	0.16 (0.12–0.22)	0.13 (0.10–0.16)	0.17 (0.13–0.21)	0.19 (0.16–0.21)	0.21 (0.17–0.25)	0.17 (0.15–0.18)
**2020**	0.18 (0.16–0.21)	0.26 (0.23–0.29)	0.23 (0.20–0.25)	0.23 (0.17–0.29)	0.11 (0.08–0.15)	0.18 (0.14–0.22)	0.26 (0.22–0.29)	0.25 (0.21–0.29)	0.21 (0.19–0.22)

Supplementary Table S2: The APC for overall AAMRs due to ATIN from 1999 to 2020.

Segment	Lower Endpoint	Upper Endpoint	APC	Lower CI	Upper CI	Prob > |t|
**1**	1999	2013	-1.8755*	-3.2056	-0.5271	0.009410
**2**	2013	2020	8.9971*	5.6879	12.4099	0.000018

* Indicates statistical significance ($p < 0.05$).

Supplementary Table S3: The APC for AAMR due to ATIN stratified by gender from 1999-2020.

Cohort	Segment	Lower Endpoint	Upper Endpoint	APC	Lower CI	Upper CI	Prob > |t|
**Female - 1 Joinpoint**	1	1999	2013	-1.5763*	-3.0991	-0.0295	0.046264
**Female - 1 Joinpoint**	2	2013	2020	9.5015*	5.8714	13.2561	0.000027
**Male - 1 Joinpoint**	1	1999	2013	-2.2316*	-3.9119	-0.5220	0.013760
**Male - 1 Joinpoint**	2	2013	2020	9.8989*	5.5996	14.3733	0.000112

* Indicates statistical significance ($p < 0.05$).

Supplementary Table S4: The APC for AAMR due to ATIN stratified by race from 1999-2020.

Cohort	Segment	Lower Endpoint	Upper Endpoint	APC	Lower CI	Upper CI	Test Statistic (t)	Prob > |t|
**White - 1 Joinpoint**	1	1999	2013	-1.2796	-2.7753	0.2392	-1.7797	0.093010

**White - 1 Joinpoint**	2	2013	2020	10.4225*	6.6993	14.2755	6.0986	0.000012
**Black or African American - 1 Joinpoint**	1	1999	2014	-4.9609*	-6.8040	-3.0814	-5.4817	0.000041
**Black or African American - 1 Joinpoint**	2	2014	2020	7.7861	-0.0194	16.2010	2.1044	0.050530

* Indicates statistical significance (p < 0.05).

Supplementary Table S5: The AAPC for different census regions AAMRs per 100000 from 1999-2020.

Cohort	Range	Lower Endpoint	Upper Endpoint	AAPC	Lower CI	Upper CI	P-Value
**Census Region 1: Northeast - 1 Joinpoint**	Full Range	1999	2020	1.6992	-0.6629	4.1175	0.159950
**Census Region 2: Midwest - 1 Joinpoint**	Full Range	1999	2020	2.3998	-0.4170	5.2963	0.095642
**Census Region 3: South - 1 Joinpoint**	Full Range	1999	2020	0.5739	-1.0289	2.2027	0.485062
**Census Region 4: West - 2 Joinpoints**	Full Range	1999	2020	2.6413	-0.5631	5.9488	0.107176

Supplementary Table S6: The APC for different census regions AAMRs per 100000 from 1999-2020.

Cohort	Segment	Lower Endpoint	Upper Endpoint	APC	Lower CI	Upper CI	Test Statistic (t)	Prob > |t|
**Census Region 1: Northeast - 1 Joinpoint**	1	1999	2013	-2.0713	-4.3904	0.3041	-1.8425	0.082912
**Census Region 1: Northeast - 1 Joinpoint**	2	2013	2020	9.6813*	3.4139	16.3286	3.3135	0.004108

**Census Region 2: Midwest - 1 Joinpoint**	1	1999	2014	-1.2328	-3.6409	1.2354	-1.0603	0.303831
**Census Region 2: Midwest - 1 Joinpoint**	2	2014	2020	12.0769*	2.9371	22.0282	2.8278	0.011606
**Census Region 3: South - 1 Joinpoint**	1	1999	2012	-3.5774*	-5.4605	-1.6567	-3.8969	0.001160
**Census Region 3: South - 1 Joinpoint**	2	2012	2020	7.7044*	4.2970	11.2230	4.8710	0.000144
**Census Region 4: West - 2 Joinpoints**	1	1999	2003	10.5706	-1.0428	23.5469	1.9422	0.072510
**Census Region 4: West - 2 Joinpoints**	2	2003	2008	-7.1728	-16.6896	3.4312	-1.4758	0.162122
**Census Region 4: West - 2 Joinpoints**	3	2008	2020	4.4084*	2.6466	6.2005	5.4368	0.000088

* Indicates statistical significance ($p < 0.05$).
